# High-throughput sequencing and degradome analysis reveal altered expression of miRNAs and their targets in a male-sterile cybrid pummelo (*Citrus grandis*)

**DOI:** 10.1186/s12864-016-2882-0

**Published:** 2016-08-09

**Authors:** Yan-Ni Fang, Bei-Bei Zheng, Lun Wang, Wei Yang, Xiao-Meng Wu, Qiang Xu, Wen-Wu Guo

**Affiliations:** Key Laboratory of Horticultural Plant Biology of Ministry of Education, Huazhong Agricultural University, Wuhan, 430070, China

**Keywords:** Citrus, miRNA, CMS, High-throughput sRNA sequencing, Degradome

## Abstract

**Background:**

G1 + HBP is a male sterile cybrid line with nuclear genome from Hirado Buntan pummelo (*C. grandis* Osbeck) (HBP) and mitochondrial genome from “Guoqing No.1” (G1, Satsuma mandarin), which provides a good opportunity to study male sterility and nuclear-cytoplasmic cross talk in citrus. High-throughput sRNA and degradome sequencing were applied to identify miRNAs and their targets in G1 + HBP and its fertile type HBP during reproductive development.

**Results:**

A total of 184 known miRNAs, 22 novel miRNAs and 86 target genes were identified. Some of the targets are transcription factors involved in floral development, such as auxin response factors (*ARFs*), *SQUAMOSA* promoter binding protein box (SBP-box), *MYB*, basic region-leucine zipper (*bZIP*), *APETALA2* (*AP2*) and transport inhibitor response 1 (*TIR1*). Eight target genes were confirmed to be sliced by corresponding miRNAs using 5’ RACE technology. Based on the sequencing abundance, 42 differentially expressed miRNAs between sterile line G1 + HBP and fertile line HBP were identified. Differential expression of miRNAs and their target genes between two lines was validated by quantitative RT-PCR, and reciprocal expression patterns between some miRNAs and their targets were demonstrated. The regulatory mechanism of miR167a was investigated by yeast one-hybrid and dual-luciferase assays that one dehydrate responsive element binding (DREB) transcription factor binds to miR167a promoter and transcriptionally repress miR167 expression.

**Conclusion:**

Our study reveals the altered expression of miRNAs and their target genes in a male sterile line of pummelo and highlights that miRNA regulatory network may be involved in floral bud development and cytoplasmic male sterility in citrus.

**Electronic supplementary material:**

The online version of this article (doi:10.1186/s12864-016-2882-0) contains supplementary material, which is available to authorized users.

## Background

Cytoplasmic male sterility (CMS), which is a maternally inherited trait in higher plants, is characterized by the inability to produce functional pollen. Due to the advantage of low cost and high efficiency in breeding, CMS has been widely used in plants to produce elite hybrid seeds, such as maize, rice and rapeseed [1–3].

CMS is known to originate from the incompatibility of nuclear-cytoplasmic interaction, a coordinate cross talk of organelle and nuclear genome. The interaction mode includes two kinds of signaling transmission patterns: signaling from nucleus to organelles, which is known as anterograde regulation, and signaling from organelles to nucleus, which is termed as retrograde (RTG) regulation [4, 5]. Chimeric novel open reading frames (ORFs) resulted from mitochondrial genome rearrangement have been proved to be involved in this retrograde signaling in CMS systems [6, 7]. It was proposed that some nuclear genes, such as homeotic floral organ identity genes, lipid transfer protein genes, basic-helix-loop-helix transcription factors, gene encoding calcium ion-dependant protein kinase, and genes responsible for premature tapetal programmed cell death (PCD), are involved in retrograde regulation process [8].

CMS is often observed in alloplasmic lines obtained from intra- or inter-specific crosses [9–11]. Somatic hybridization allows the production of new germplasm by fusion of protoplasts from both mesophyll parent and callus parent, which overcomes the difficulties in traditional citrus breeding, such as sexual incompatibility, nucellar polyembryony, and pollen/ovule sterility [12, 13]. Many citrus cytoplasmic hybrids with nuclear genome from leaf parent and mitochondrial DNA from callus parent have been obtained through symmetric fusion, which allows the transfer of mitochondria-controlled specific agronomic traits [14–17]. Using this method, a cybrid line G1 + HBP with mitochondrial genome from “Guoqing No.1” (G1, Satsuma mandarin) and nuclear and chloroplast genomes from Hirado Buntan pummelo (*C. grandis* Osbeck) (HBP) was regenerated [16]. G1 + HBP is an elite citrus cultivar with seedless phenotype, and can be considered as a male sterile mutant of HBP with cytoplasmic genome substitution [18, 19]. Thus, the cybrid line G1 + HBP provides a good opportunity to study the traits arising from nuclear-cytoplasmic cross talk, particularly male sterility. In a previous research, some genes which involved in nucleic acid binding and response to hormone synthesis and metabolism were shown to be differentially expressed in transcriptomics and proteomics analysis [20]. However, it was found that the correlation coefficient between the transcriptome and proteome was very low, suggesting that post-transcriptional regulation such as that from miRNA, may be involved in the process [20].

miRNAs are a class of endogenous non-coding RNAs of ~22 nt in length, which are excised from stem-loop structures of double-strand RNA precursors by Dicer enzyme [21–23]. In plants, miRNAs can regulate protein-coding genes expression post-transcriptionally via mRNA cleavage or translational repression. miRNAs were confirmed to be involved in various biological processes during plant development, including tissue development, floral organ identity, flowering phase transition, auxin signal transduction and stress response, etc. [24]. In addition, the involvement of miRNAs in CMS has also been reported in some species. Eight miRNAs in maize exhibited considerable differences between the CMS line and the fertile line, suggesting that miRNAs participate in maize CMS [25]. Another research in *Brassica juncea* also proposed that miRNAs are associated with CMS, as differential expression of miRNAs was observed when comparing the CMS line with its fertile line [26]. *PPR* (pentatricopeptide repeat) genes, the main nuclear restorer-of-fertility genes in CMS phenotype, have been identified to be targeted by miRNAs [27, 28]. Several studies have revealed the important roles of miRNAs in citrus stress response [29], fruit development [30] and somatic embryogenesis [31]. However, there has been no report about the roles of miRNAs in nuclear-cytoplasmic communication in citrus CMS.

In this study, a comprehensive comparative analysis of miRNAs was performed between a fertile line HBP and a male sterile cybrid line G1 + HBP by high-throughput sequencing. Global change of miRNA abundance was investigated and the roles of miRNAs in cytoplasmic male sterility were elucidated. To dissect the regulatory relationship between miRNA and CMS in citrus, the targets of miRNA were identified through degradome sequencing and the altered expression of miRNAs and their target genes in G1 + HBP compared with in HBP was demonstrated.

## Results

### Overview of the sRNA sequencing

**Fig. 1:**
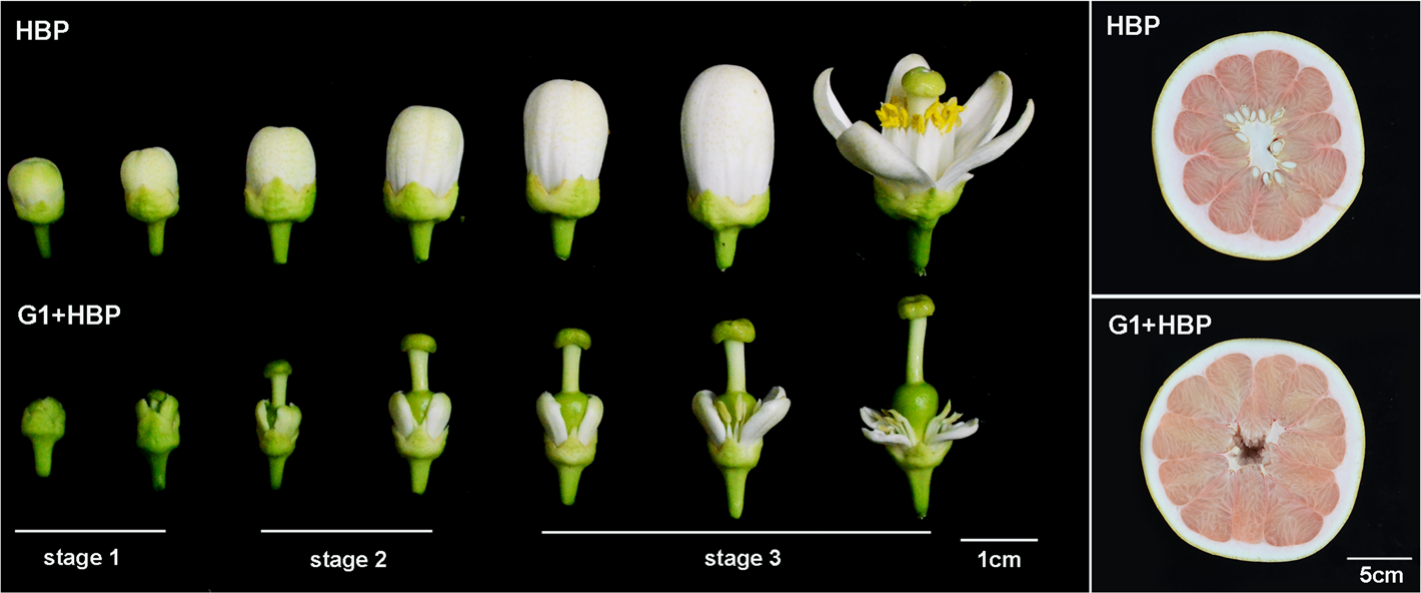
Phenotype of flower buds and fruit samples of HBP (above) and G1 + HBP (below) at three stages

Twelve libraries comprising three stages of HBP (male fertile and seedy type) and G1 + HBP (male sterile and seedless type) (Fig. 1) were constructed and sequenced, yielding respectively an average of 18 million and 39 million raw reads per sample in the two replicates, respectively. After removing the low quality reads, 3’adapter, and those tags smaller than 18 nt, clean reads with length ranging from 18 to 30 nucleotides were obtained, accounting for approximately 99 % of the total reads (Table 1).

**Table 1:** Summary of small RNA sequencing data and annotation after alignment to GenBank and Rfam in twelve libraries

Sample	HB-1		HB-2		HB-3		GH-1		GH-2		GH-3	
Replicates	HB-1_1	HB-1_2	HB-2_1	HB-2_2	HB-3_1	HB-3_2	GH-1_1	GH-1_2	GH-2_1	GH-2_2	GH-3_1	GH-3_2
Total reads	17037715	38683099	17621481	39548578	19663044	40162945	17535885	42963064	15705025	44430802	15566886	44635615
High quality	16999950	38487499	17584242	39343421	19623323	39952352	17500038	42748962	15674392	44207130	15531635	44414589
Clean reads	16956969	38325616	17545718	39013183	19532324	39667678	17269743	42429457	15616800	43371288	15486371	44193060
	(99.5 %)	(99.1 %)	(99.6 %)	(98.6 %)	(99.3 %)	(98.8 %)	(98.5 %)	(98.7 %)	(99.4 %)	(97.6 %)	(99.5 %)	(99.0 %)
Unique reads	6269391	11867928	6312996	10786793	6962669	10862638	6093498	12676590	5838882	12526797	5611881	12624626
Exon_antisense	167765	301351	161911	302793	182564	279824	182182	327182	154665	314229	146167	311287
	(2.68 %)	(2.54 %)	(2.56 %)	(2.81 %)	(2.62 %)	(2.58 %)	(2.99 %)	(2.58 %)	(2.65 %)	(2.51 %)	(2.60 %)	(2.47 %)
Exon_sense	270529	490497	251688	459169	267032	439731	276961	520264	236032	495847	225372	471758
	(4.32 %)	(4.13 %)	(3.99 %)	(4.26 %)	(3.84 %)	(4.05 %)	(4.55 %)	(4.1 %)	(4.04 %)	(3.96 %)	(4.02 %)	(3.74 %)
intron_antisense	66416	115941	66338	102366	75012	103434	65927	123923	62673	119586	60638	122622
	(1.06 %)	(0.98 %)	(1.05 %)	(0.95 %)	(1.08 %)	(0.95 %)	(1.08 %)	(0.98 %)	(1.07 %)	(0.95 %)	(1.08 %)	(0.97 %)
intron_sense	86296	144496	84417	128534	96497	130467	87253	154342	81847	151772	79880	155531
	(1.38 %)	(1.22 %)	(1.34 %)	(1.19 %)	(1.39 %)	(1.2 %)	(1.43 %)	(1.22 %)	(1.40 %)	(1.21 %)	(1.42 %)	(1.23 %)
rRNA	76868	95598	59951	90644	62681	90479	69226	84718	57901	98165	65986	91555
	(1.23 %)	(0.81 %)	(0.95 %)	(0.84 %)	(0.90 %)	(0.83 %)	(1.14 %)	(0.67 %)	(0.99 %)	(0.78 %)	(1.18 %)	(0.73 %)
snRNA	2494	4615	2051	5184	2258	4792	2807	4981	2084	5499	2205	5476
	(0.04 %)	(0.04 %)	(0.03 %)	(0.05 %)	(0.03 %)	(0.04 %)	(0.05 %)	(0.04 %)	(0.04 %)	(0.04 %)	(0.04 %)	(0.04 %)
snoRNA	953	2034	930	1741	882	1663	986	2200	811	2161	818	1966
	(0.02 %)	(0.02 %)	(0.01 %)	(0.02 %)	(0.01 %)	(0.02 %)	(0.02 %)	(0.02 %)	(0.01 %)	(0.02 %)	(0.01 %)	(0.02 %)
tRNA	8148	9972	6239	9656	6846	10974	7579	10219	6224	13986	8251	15950
	(0.13 %)	(0.08 %)	(0.10 %)	(0.09 %)	(0.10 %)	(0.10 %	(0.12 %)	(0.08 %)	(0.11 %)	(0.11 %)	(0.15 %)	(0.13 %)
unann	5555591	10664812	5648863	9650918	6235482	9764013	5367007	11405633	5204483	11282120	4996366	11407309

**Fig. 2:**
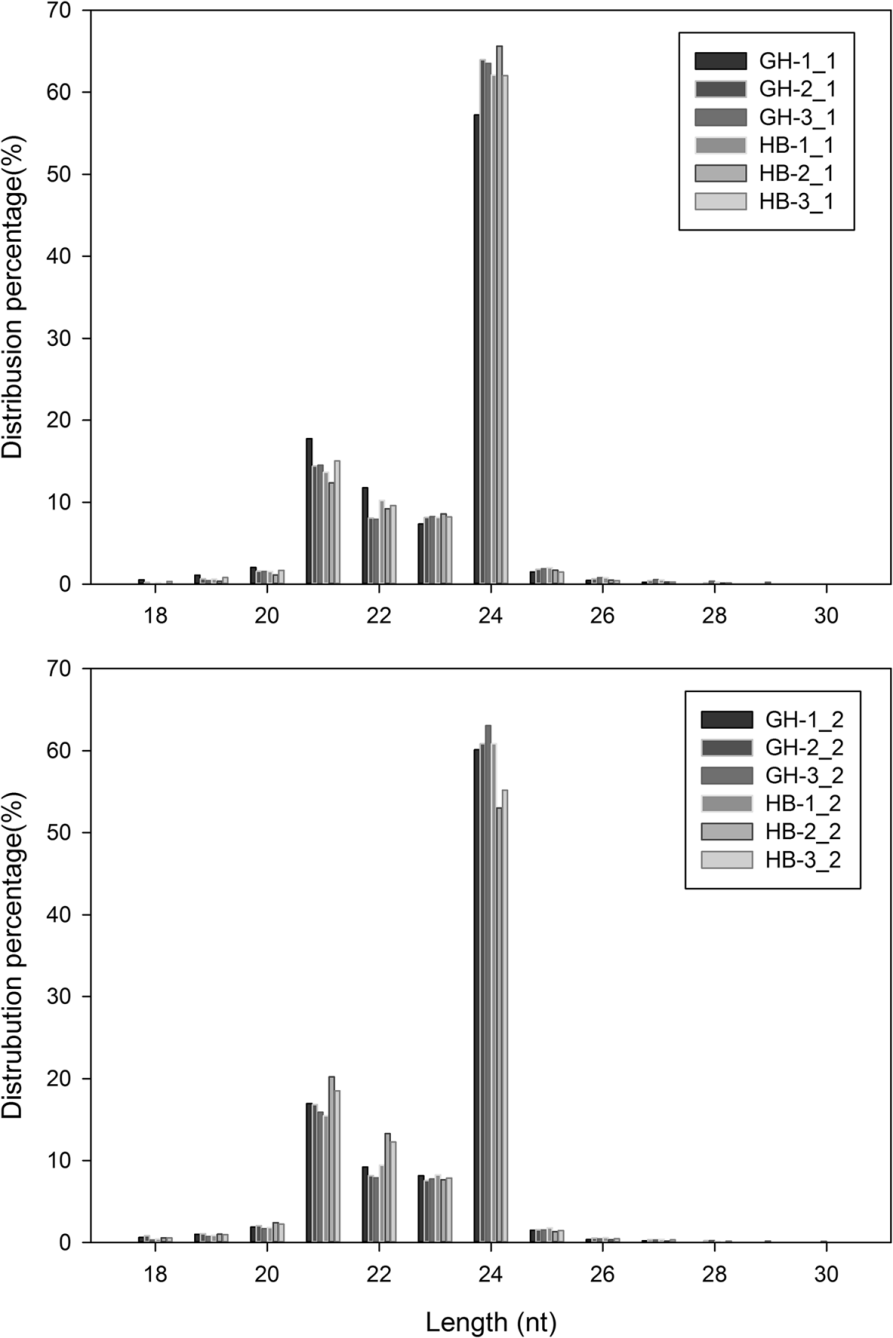
Length distributions of small RNAs in the twelve libraries for two replicates. The 21–24 nt account for the majority of the reads and 24 nt is the largest group

In length distribution analysis, the majority of the reads were 21–24 nt (Fig. 2), which is consistent with the result in other species [32, 33]. The 24 nt sRNAs were the most abundant, accounting for more than half of the total reads, followed by 21 nt sRNAs, though the number was much smaller than that of 24 nt sRNAs. From Fig. 2, it could be observed that the length distribution of sRNAs was very similar between HBP and G1 + HBP at the three stages. Afterwards, the sRNAs were analyzed by BLASTN against other known non-coding RNAs deposited in the Rfam and NCBI database to annotate different kinds of sRNAs. All of the twelve libraries showed nearly identical compositions of different non-conding RNA types, including rRNA (~1 %), tRNA (~0.1 %), snoRNA (~0.02 %) and snRNA (~0.04 %). The detailed data for each library are shown in Table 1. The remaining sequences were examined to identify miRNAs, and the un-annotated sequences were used for novel miRNAs identification.

### Identification and different expression of miRNAs

**Fig. 3:**
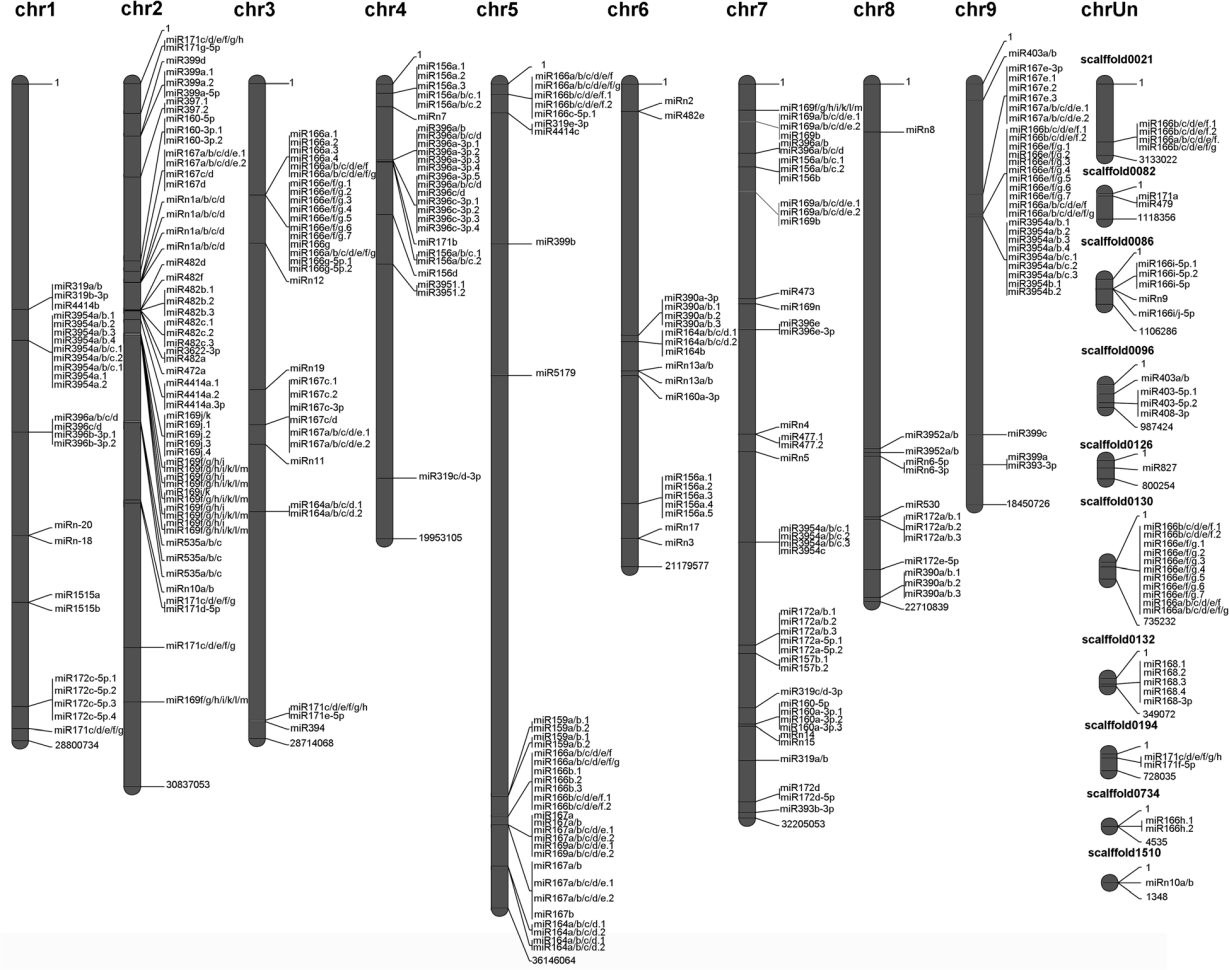
Location of 184 known miRNAs and 22 novel miRNAs on the 9 chromosomes of sweet orange. Some miRNAs were located in some regions which were un-annotated (chrUn)

We mapped the unique sRNAs into miRBase 20 to identify the known miRNAs based on the criteria of mismatch ≤ 2. As a result, 184 known miRNAs belonging to 36 families were identified (Additional file 1). Among the 36 families, 27 were conserved in many plant species, including both eudicot and monocot, and nine were non-conserved, which were found in only one or a few plant species. miRNAs were mapped onto the genome sequences of *Citrus sinensis* to discover their precursors and analyze their locations on the chromosomes. As a result, 115 precursors were discovered (Additional file 2). A figure representing the mapping result was generated using the joinmap4.1 software (Fig. 3). From this figure, it can be observed that for some precursors, one single precursor could yield multiple miRNAs, such as the precursors of MIR166 and MIR3954; and for some miRNAs, one single miRNA might originated from four to six different precursors, such as cga-miR164a/b/c/d.1, cga-miR166a/b/c/d/e/f and cga-miR167a/b/c/d/e.1. The miRNAs originating from the same precursor were termed isomiRs [34]. In our libraries, cga-miR3954a, whose function was unknown, corresponded to up to nine isomiRs. Taken together, the location analysis demonstrated the complexity of the origin of miRNAs.

The un-annotated sRNA sequences were used to identify the citrus specific miRNAs. After mapping the sRNAs to the sweet orange genome, we used MIREAP software to analyze their secondary structure. Based on the criteria previously established [23, 35], 22 novel miRNAs were newly identified to be specific to citrus (Additional file 2).

**Fig. 4:**
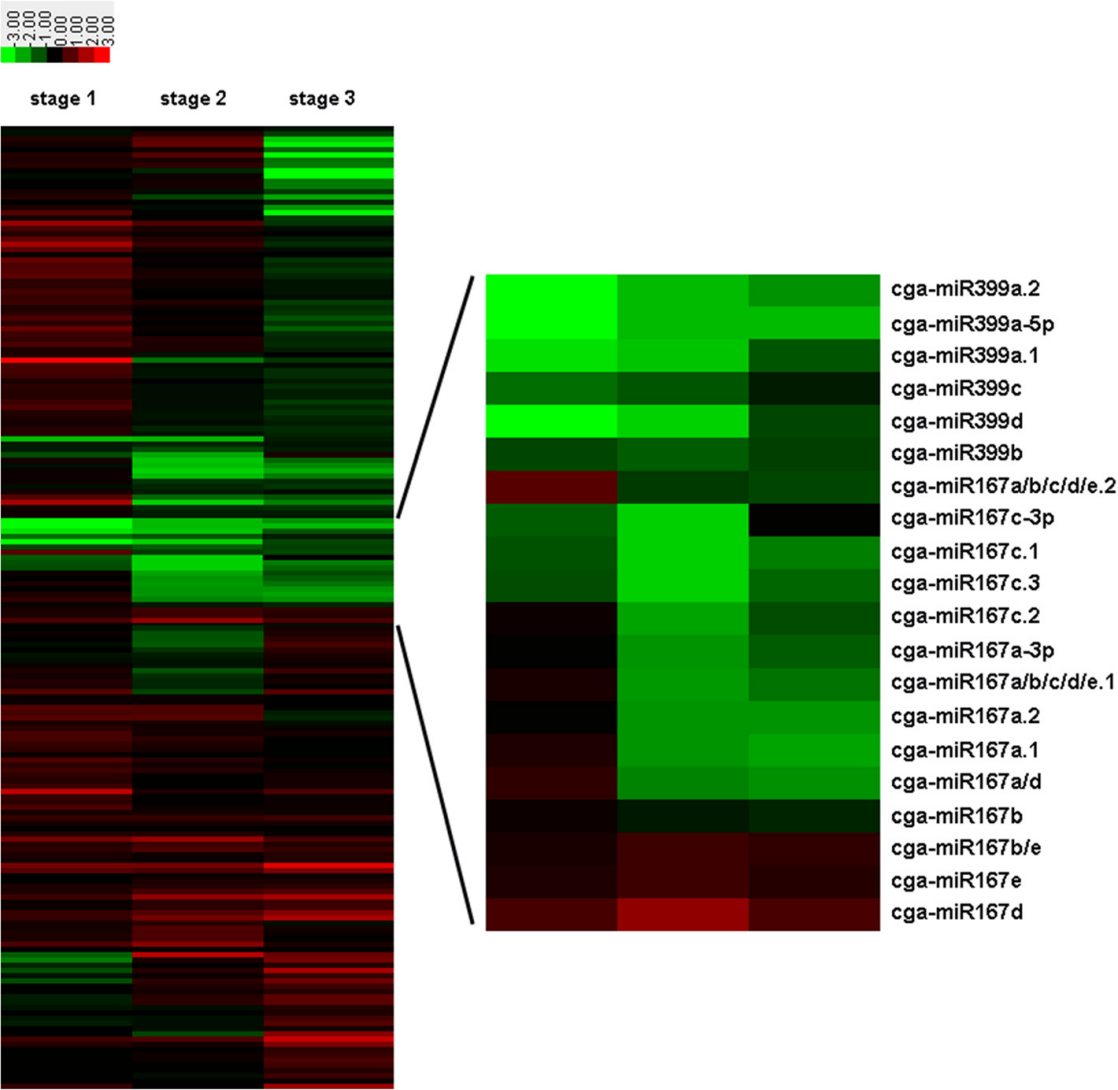
Cluster heat map of expression of miRNAs. The expression of 184 known miRNAs between HBP and G1 + HBP and all of the members in miR167 and miR399 at three stages in sequencing data was shown. The ratio value was log2 transformed for each miRNA. Green indicates down-regulation pattern and red indicates up-regulation pattern

**Fig. 5:**
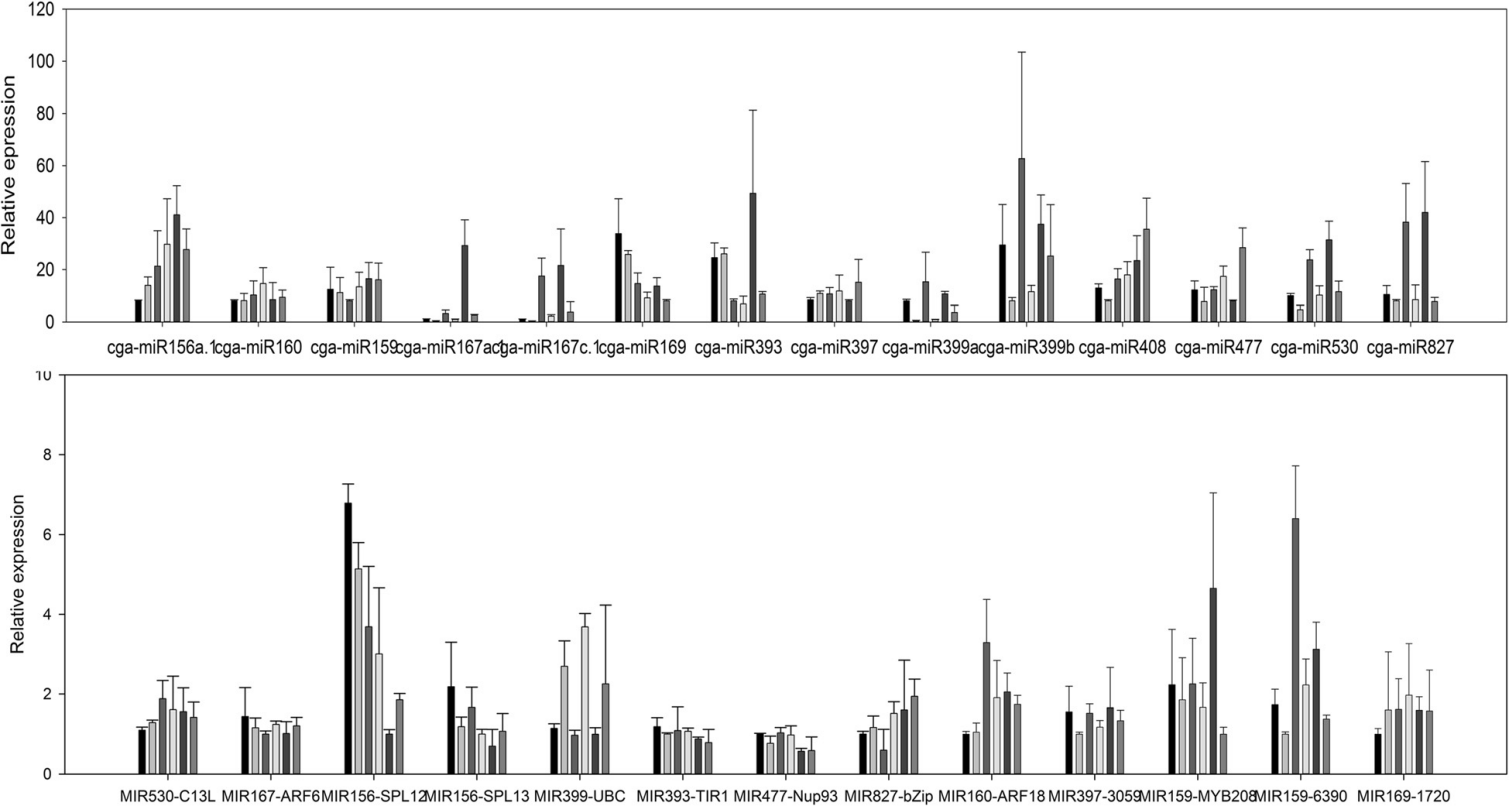
qRT-PCR results of 14 miRNAs and 13 target genes between HBP and G1 + HBP at three stages. X-axis indicates miRNAs and targets. The six columns on the X-axis refer to HB-1, G1 + HB-1, HB-2, G1 + HB-2, HB-3, G1 + HB-3, respectively. Y-axis indicates the relative expression in HBP and G1 + HBP. Columns and bars represent the means and standard errors (*n* = 4)

**Fig. 6:**
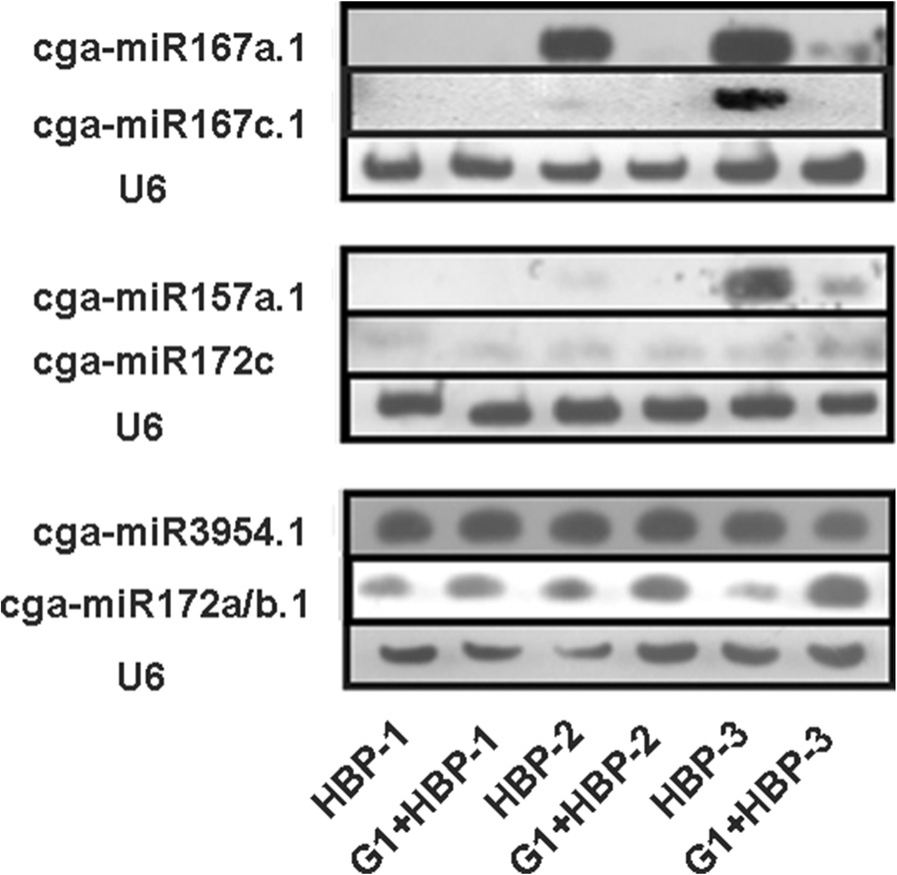
Northern blot analysis of 6 miRNAs in HBP and G1 + HBP at three developmental stages. U6 acts as the control

To ensure the reliability of the data, 12 libraries which included two biological replicates of HBP and G1 + HBP at three floral developmental stages were constructed for sRNA sequencing. After normalizing the count of miRNAs into transcript per million (TPM) which was then log (ln) transformed, six scatter diagrams reflecting the correlation of the normalization data between the two biological replicates for all of the six samples were generated. As expected, the sequencing data of miRNAs in two biological replicates showed good repeatability (*r* = 0.85–0.99) (Additional file 3), demonstrating the results were reliable. To identify the differentially expressed miRNAs between HBP and G1 + HBP, the expression abundance of all the known miRNAs at three stages was compared using Student’s *T*-test based on the filter parameter of fold change >1.5 and FDR for Student’s *t*-test <0.05 after being subjected to TPM normalization. As a result, a total of 42 miRNAs showed differential expression between HBP and G1 + HBP (Additional file 1). A heat map was generated by hierarchical (average linkage) clustering based on the expression patterns in G1 + HBP versus HBP at three stages [36] (Fig. 4). From this figure, we found that almost all of the members of miR167 family, especially those originating from the precursor pre-miR167a (cga-miR167a.1, 167a.2, 167a-3p, 167a/d, 167a/b/c/d/e.1, 167a/b/c/d/e.2), showed similar expression patterns and were significantly down-regulated (>3 fold) at stage 2 and 3 (Fig. 4). All the members in miR399 family were down-regulated during the whole reproductive process. The expression profiles of 14 known miRNAs were validated using stem-loop RT-PCR technique (Fig. 5). The results of qRT-PCR for most of the miRNAs were in agreement with those of the sequencing data (Additional file 3). Besides, the qPCR result for each biological replicate showed high reproducibility (Additional file 3), demonstrating good reliability of differential expression result. Northern blot analysis further validated the expression of six miRNAs, which is consistent with the result of deep sequencing as expected (Fig. 6).

### Comprehensive targets identification by degradome sequencing

One degradome library was constructed with balanced mix of RNAs in six samples. The sequencing yielded 21,622,689 total reads, among which 21,541,352 (99.6 %) were clean reads after removing the low quality reads, 5’ and 3’ adapter contaminants and the reads smaller than 18 nt. These clean reads were comprised of 1,971,972 unique reads as indicated by initial analysis. When mapping the unique reads to sweet orange genome, we found that 1,398,670 unique reads (70.9 %) had perfect match to the genome. After searching in the NCBI genbank and the Rfam database, a small number of them (0.26 %) were annotated as non-coding RNAs like rRNA, tRNA, snRNA and snoRNA. Most of the unique reads (66.7 %) could be mapped onto the sense and antisense strands of cDNAs. The data of degradome sequencing are detailed in Table 2. Based on the regulatory mode of miRNA to mRNA, the tags which could be mapped onto the cDNAs were further analyzed to identify miRNA targets.

**Table 2:** Degradome data summary for mixed samples with six materials

	Total reads	Unique reads
Clean reads	21541352	1971912
Mapped to the genome	9283181	1398670
cDNA_antisense	69356(0.32 %)	15559(0.79 %)
cDNA_sense	8718631(40.47 %)	1300884(65.97 %)
rRNA	16851(0.08 %)	1820(0.09 %)
snRNA	6768(0.03 %)	1792(0.09 %)
snoRNA	6633(0.03 %)	1311(0.07 %)
tRNA	995(0.00 %)	291(0.01 %)
Unann	12722118(59.06 %)	650255(32.98 %)

**Fig. 7:**
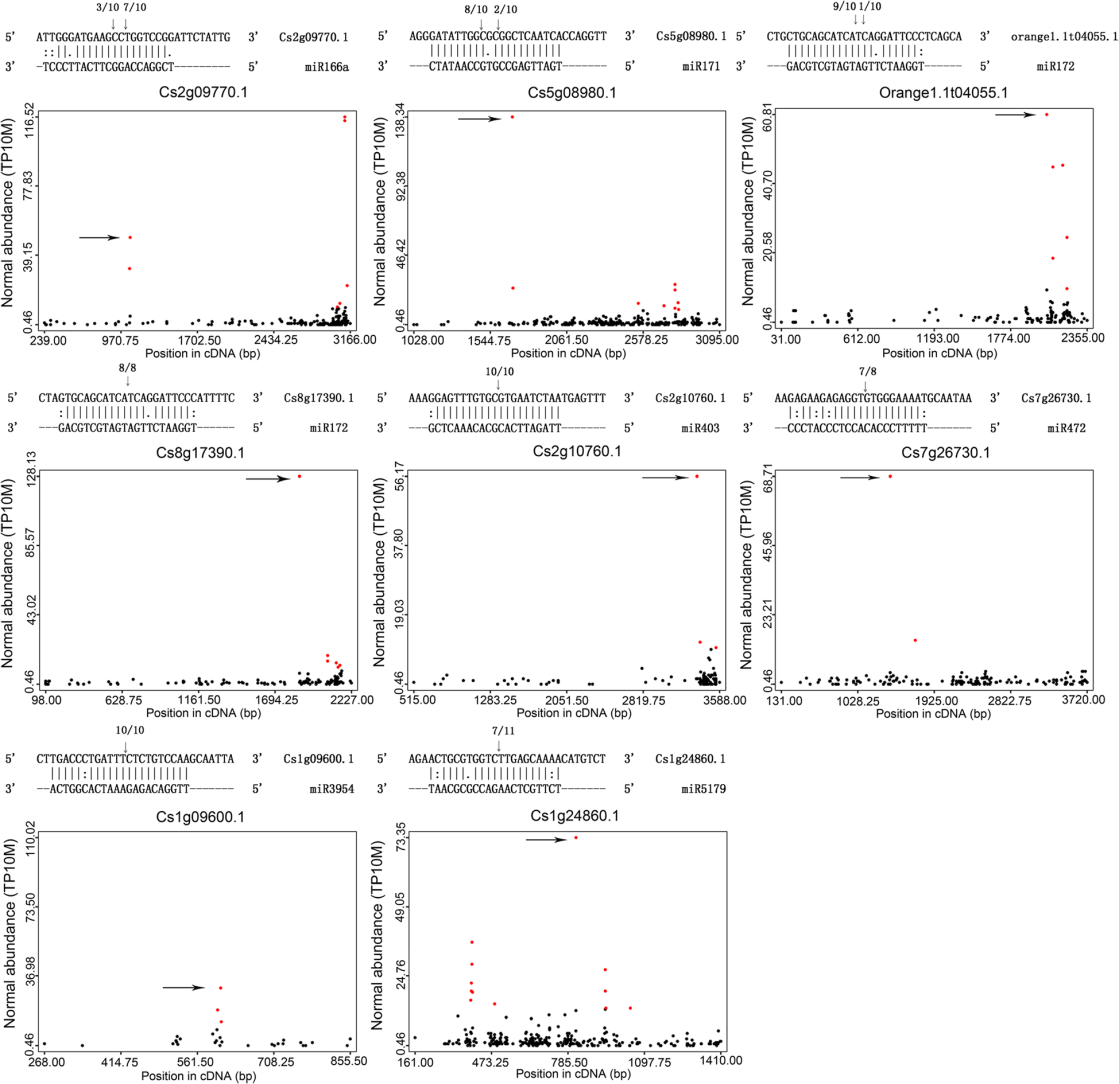
Validation of 8 miRNA target genes by RLM 5’-RACE. The arrows mark the cleavage site and the numbers indicate the frequency of cloned PCR products in corresponding cleavage sites

A total of 86 targets for 39 miRNAs (31 known miRNAs and eight novel miRNAs) were identified (Additional file 4). The targets were categorized into three classes based on their abundance of miRNA-aligned tags among the transcripts [37]. The targets were classified into class 1 if the degradome tags indicative of miRNA-mediated cleavage were the most abundant tags matching the transcript, such as the transcription factors such as SBP-box, AP2, MYB, GRAS, NAC and ARF. These targets were conserved in plants. For example, miR156 and miR167 targeted 3 *SPL* genes and 2 *ARF* genes respectively, which are in agreement with the results in other plant species, such as *Arabidopsis* [38], apple [39] and tomato [40]. Almost all the targets of novel miRNAs belonged to class 3, which contained the target genes with very low abundance of miRNA targeted tags. Some genes, such as miR162, miR168 and miR403, which were involved in the biogenesis of miRNAs, were also found to be miRNA targets. miR162 targeted one Dicer like 1 enzyme (DCL1), which is one of the main enzyme for processing pre-miRNA and mature miRNA [22]. miR403 targeted AGO2 (Argonaute protein) and miR168 targeted AGO1, while AGO proteins are responsible for loading miRNA guiding strand to form the core of miRNA induced silencing complexes (miRISCs) and repressing genes transcription through targets slicing [41]. Some miRNAs targeted putative non-coding RNAs, such as miR3954, miR390, miR3951 and miRn-12. In addition to the targets mentioned above, some miRNAs targeted some genes that encode resistance proteins, such as miR472, miR482 and miR535. Eight cleavage targets identified in the degradome sequencing analysis were satisfyingly verified by RLM-5’ RACE technique, which further confirmed the reliability of the degradome data (Fig. 7).

### Gene ontology enrichment and expression analysis of mRNA targets for differentially expressed miRNAs

**Fig. 8:**
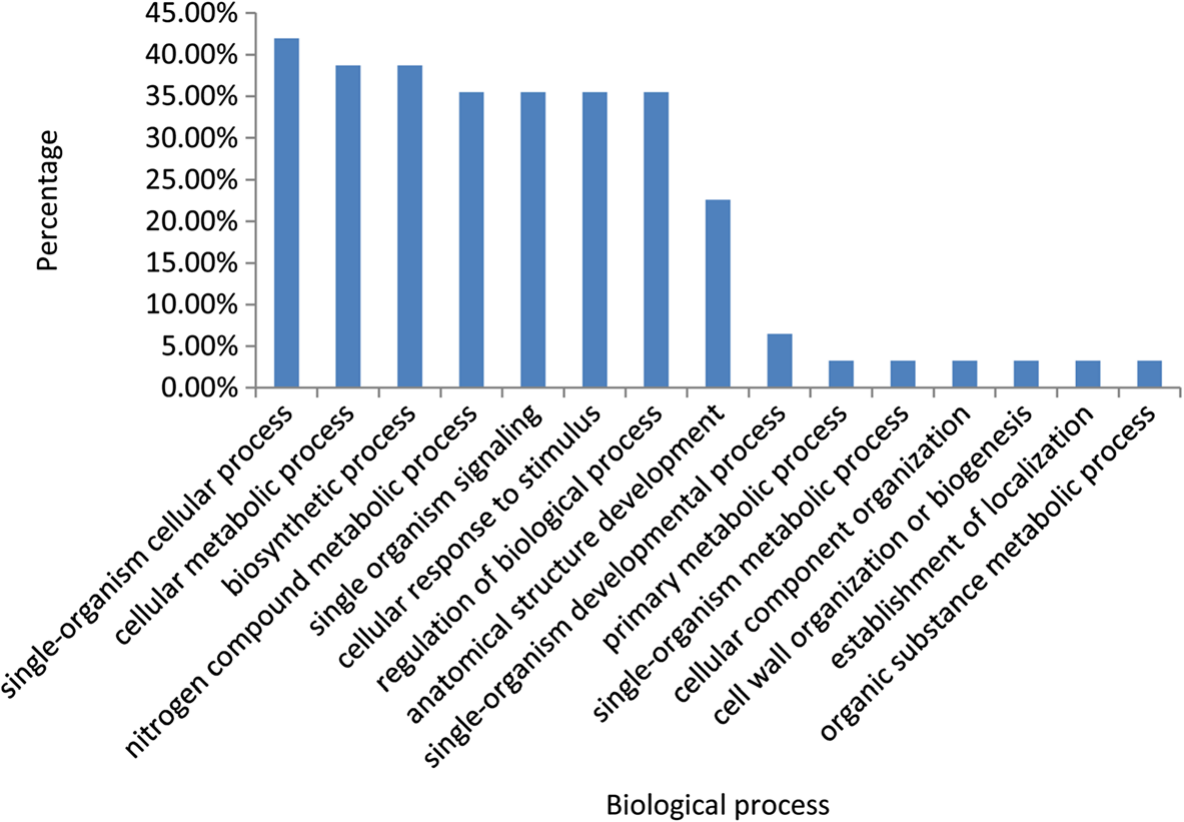
GO enrichment analysis of 42 targets genes of differentially expressed miRNAs

Forty two genes were identified to be the targets of differentially expressed miRNAs. Blast2go program (https://www.blast2go.com/) was applied for enrichment analysis and functional annotation of these genes to figure out the biological processes they are involved in [42]. The analysis indicated that these target genes participate in various biological processes, such as cellular metabolic process, biosynthetic process, response to stimulus, signaling, anatomical structure development, cell wall organization or biogenesis (Fig. 8).

Thirteen targets of fourteen known miRNAs were selected to validate their expression difference, using two biological replicates and four technological repeats (Fig. 5). The statistical data on differences of miRNAs and target genes were detailed in additional file 3. Consistent with the sequencing data, miR167, 393, 399, 530 and 827 were all significantly down-regulated in G1 + HBP, especially at stage 3, suggesting their important role in reproductive development. Cga-miR477 was down-regulated at stage 1 but up-regulated at stages 2 and 3. Cga-miR156a.1 was up-regulated at stages 1 and 2 but down-regulated at stage 3. As the targets of miR156a, *SPL12* and *SPL13* showed exactly opposite expression pattern with cga-miR156a.1, which decreased at the first two stages and increased at the third stage. All the miR399 members were significantly down-regulated (>2 fold), and the expression of *UBC/PHO2* was increased in G1 + HBP, which was opposite from that of miR399. miR827 was also obviously inhibited in G1 + HBP, especially at stages 2 and 3. And as the target of miR827, a bZIP transcription factor was slightly up- regulated. However, the targets of miR167, 477 and 530 were not regulated, which did not show obvious change in G1 + HBP during flower development.

### DREB binds to the miR167a promoter and represses miR167a expression

**Fig. 9:**
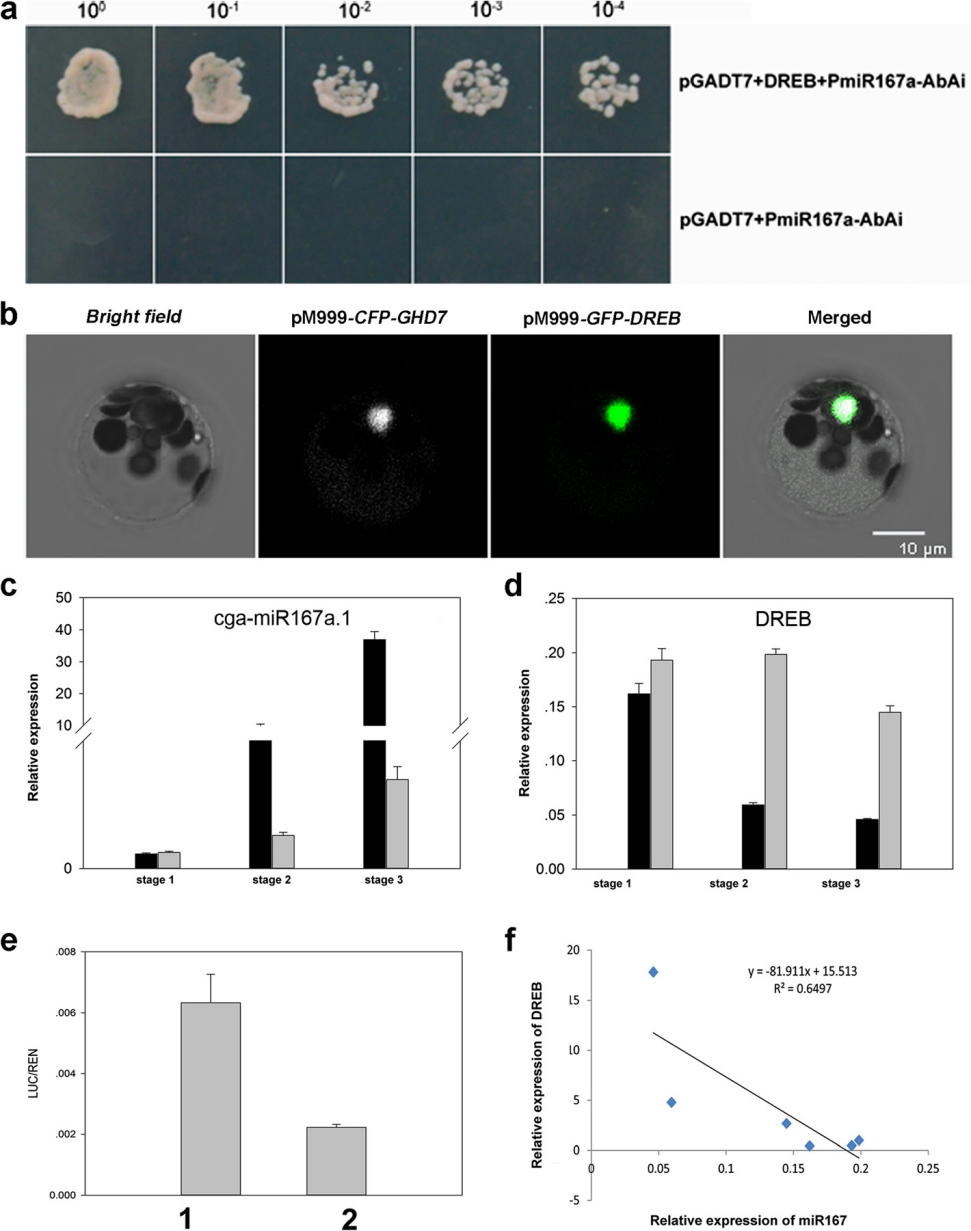
Relationship and regulation of DREB transcription factor to miR167 by yeast one-hybrid and dual-luciferase assay and qPCR analysis. **a** Yeast one-hybrid assay. **b** Subsellular localization of DREB transcription factor. **c** qPCR analysis of cga-miR167a.1. (d) qPCR analysis of DREB transcription factor. **e** Dual-luciferase assay. 1, pGReenII 0800-PmiR167a::LUC/pH7GW2D-DREB; 2, pGReenII 0800-PmiR167a::LUC/pH7GW2D. **f** The correlation analysis between the expression pattern of cga-miR167a.1 and DREB in six samples

Almost all of the members in miR167 family showed similar expression patterns and were significantly down-regulated in G1 + HBP, suggesting that miR167 members might play important roles in citrus floral bud development and CMS with a common regulatory mechanism. Pre-miR167a was considered as one of the main precursors, for it generate the most abundant mature miR167. In order to understand the regulatory mechanism of miR167a in citrus floral development, yeast one-hybrid assay was conducted to identify the upstream regulators of miR167a. First, an in silico analysis of the promoter region of miR167a in HBP and G1 + HBP were performed by the cis-element PLACE database (https://sogo.dna.affrc.go.jp/cgi-bin/sogo.cgi?sid=&lang=en&pj=640&action=page&page=newplace) and plantCARE database (http://bioinformatics.psb.ugent.be/webtools/plantcare/html/). The promoter sequences of HBP and G1 + HBP showed no difference and contained several cis-acting regulatory elements, such as ethylene-responsive element, G-box, gibberellins-responsive element, MYB binding site. One binding cite for dehydrate responsive element (DRE) (ACCGAC), was also found in the promoter region. The promoter sequence of miR167a was used as a bait to screen all of the transcription factors in the cDNA library using yeast one-hybrid approach. As a result, one DREB transcription factor was found to bind to the miR167a promoter (Fig. 9a). Dual-luciferase assay of transiently transformed *Nicotiana benthamiana* leaves was applied to investigate how DREB transcription factor regulates the expression of miR167a. From Fig. 9e, the analysis revealed that DREB negatively regulates the expression of miR167a. The expression abundance analysis of *DREB* gene indicated that *DREB* gene was up-regulated in G1 + HBP, which is opposite to the expression pattern of miR167a (*r* = −0.8) (Fig. 9f), confirming the negative regulatory relationship between *DREB* and miR167a. The subcellular localization of DREB protein was also assayed. A vector, *35 s:DREB:GFP*, was constructed and GHD7 was fused to CFP as a nuclear protein marker. The result showed that DREB was co-localized with CFP-fused GHD7 in the nucleus, suggesting that DREB is a nuclear protein (Fig. 9b).

## Discussion

### Identification of miRNAs and their targets by high throughput sequencing

The development of high throughput sequencing technology and completion of the genome sequencing of citrus in recent years have greatly advanced the biological research on miRNAs. In our study, 184 known miRNAs of 36 miRNA families and 22 novel miRNAs and 86 targets were identified in HBP and G1 + HBP, covering three floral and reproductive developmental stages in two years. In addition, despite the redundant sequences, 115 precursors were predicted after mapping the miRNAs into the sweet orange genome (*Citrus sinensis* Osbeck). Pummelo and sweet orange shared similar genome sequences as they are within the same citrus genus. Besides, it was suggested in a previous study that the sweet orange is an interspecific hybrid between pummelo and mandarin [43]. Hence, sweet orange genome can be applied as reference genome to analyze the miRNAs. Consistent with the report in other species [44], many miRNA variants (isomiRs) were detected, which had one to two nucleotides shift for each other on a same precursor.

Degradome sequencing technology has been widely applied in many species to identify miRNA targets in a large scale [45–47]. In our study, one library with mixed samples, including six materials which were used for sRNA sequencing, was constructed to identify the cleaved targets of miRNAs. Analysis of the targets showed that most of them are transcription factors which are conserved among plant species. Some target genes, including *ARF*, *MYB*, *AP2*, *TM6*, *bZIP* and *F-box*, have long been shown to be associated with plant floral or reproductive development. Those genes encoding uncharacterized proteins or some ncRNAs could be regarded as good resources to be integrated into the regulatory network of miRNAs in male sterility or in citrus development in future research. Eight genes were validated to be targets of corresponding miRNAs by RLM-5’ RACE technique, which is consistent with the degradome data.

### Several miRNA regulatory networks might be involved in the retrograde regulation

It is widely accepted that the function of plant cell depends on the coordination of plastid, mitochondrial and nuclear genomes. CMS is known to result from organellar-nuclear incompatibility when mitochondrion genome from one species is combined with the nuclear genome from another species [8, 48]. CMS phenotype was frequently observed in the alloplasmic lines derived from somatic fusion or interspecific crosses [49]. The cybrid line G1 + HBP in our study, which was produced by somatic hybridization, exhibited a typical male sterility phenotype and thus is an elite material to study nuclear-cytoplasmic cross talk which can result in CMS. CMS phenotype caused by cytoplasmic substitution is associated with signaling from mitochondrion to the nucleus, which is called retrograde regulation [4]. The nuclear genes change their expression as target genes in response to the mitochondrial signaling, which subsequently affects male gametophyte development [50]. Some nuclear genes encoding MADS-box class proteins, protein kinases and bHLH transcription factor have been speculated to be possible targets in retrograde regulation [8]. In our study, the male sterile cybrid line exhibited altered expression of miRNAs and their targets genes, demonstrating important roles of miRNAs regulation network in the retrograde signaling in CMS.

In the expression data of miRNAs, 42 miRNAs showed significant difference between male fertile line and male sterile line, suggesting that miRNAs play important roles in citrus male gametophyte development. GO enrichment analysis on the targets of differentially expressed miRNAs indicated that they are involved in various biological processes, such as cellular metabolism, signaling, stimulus response, anatomical structure development and cell wall organization processes. In the expression analysis of selected nuclear target genes, most of the targets showed differential expression between HBP and G1 + HBP. Almost half of the target genes were negatively regulated by corresponding miRNAs, especially miR156 (*r* = −0.96) and miR399 (*r* = −0.88). The reason why some miRNAs like miR167 and miR530 did not show negative regulation to their targets might be that they function at the translational level.

In the past decade, the roles of miRNAs in reproductive development have been well studied in *Arabidopsis*. miR156 and miR159 have been reported to be related to male sterility through targeting SBP-box genes, *GAMYB33/65*, respectively [51]. Over expression of miR156 in a *spl8* mutant background was reported to result in fully male sterile *Arabidopsis* [52]. Based on the expression analysis of miRNAs and their corresponding targets, miR156-SPL, miR167-ARF, miR399-UBC, miR827-bZIP regulatory network were selected as four candidate miRNAs-targets combinations that might be involved in citrus CMS. In our previous research in a male sterile type Ponkan mandarin (*Citrus reticulata*) [36], miR156 was shown to play an important role in anther development by negatively regulating *SPL9* and *SPL13*. In this study, miR156a negatively regulated SBP-box genes and displayed differential expression between HBP and G1 + HBP, indicating that miR156-SPL regulatory network may participate in the male sterility. Basic leucine zipper (*bZIP*), a transcription factor gene which is the target of miR827, plays pivotal roles in various biological processes including flower development, stress signaling and pathogen defense [53]. It is reported in that a bZIP transcription factor bZIP34 regulates pollen development of *Arabidopsis* by participating in pollen wall patterning [54]. bZIP transcription factor plays an important role in floral architecture and reproductive organ development through binding to a MADS-box transcription factor AGAMOUS (AG) [55]. Down-regulation of miR827 resulted in decreased expression of *bZIP*, which might be related to the defective organ development in the cybrid line G1 + HBP.

In our analysis, miR399 targeted a *UBC/PHO2* gene that encods E2 enzyme, which was consistent with the result in *Arabidopsis* [56]. miR399 has been shown to be involved in plant responses to phosphate starvation by targeting a *PHO2/UBC* gene which functions in phosphate accumulation [56]. In a previous research on rice, a *pho2* mutant exhibited defects in reproductive development during the whole growing period since inorganic phosphate is an essential component for plant propagation [57]. The expression of all of the miR399 members was significantly repressed in cybrid line G1 + HBP. In another research in Ponkan (*Citrus reticulata*), miR399 was significantly down-regulated in male sterile line [36]. miR399 and *UBC/PHO2* may participate in the male sterility phenotype in G1 + HBP through affecting phosphate (Pi) homeostasis.

miR167 has been confirmed to function in *Arabidopsis* and tomato male reproductive development by regulating the hormone pathways [58]. In our study, miR167 targeted ARF genes, which are involved in the auxin regulatory network. The auxin level in HBP and G1 + HBP showed significant difference (Additional file 5), mimicking the result of *Brassica juncea* that the nuclear genes modulated by nuclear-cytoplasmic incompatibility alter auxin response in cytoplasmic male sterile line [59]. From the significant decline of miR167 expression, we speculate that some upstream factors may be involved in the miRNA regulatory pathway in the retrograde regulation through mediating retrograde signals. As a result, by yeast one-hybrid and tobacco transient expression of a dual luciferase assay, a DREB transcription factor was found to bind to the promoter of miR167 and inhibit the activity of the promoter of miR167 transcriptionally. DREB transcription factor was previously elucidated to play an important role in plant stress response, including oxidative stress, cold resistance and drought resistance [60]. The qPCR analysis confirmed that the expression pattern of miR167 in the six libraries was negatively correlated with that of DREB transcription factor (*r* = −0.8) (Fig. 9f). DREB might act as a transcription repressor of miR167 and inhibit its expression in CMS cybrid line. A previous study has also reported that organelle will send signals, like nuclear-encoded alternative oxidase (AOX) to the nucleus to coordinate nuclear and organelle activities when nuclear and organelle genomes are integrated into one cell [61]. A DREB subfamily transcription factor was identified as a target gene of retrograde regulation and it plays an important role in mediating MRR signals to induce the expression of *AOX1a* in *Arabidopsis* [59]. Increased expression of DREB and decreased expression of miR167 in cybrid line G1 + HBP demonstrated that DREB-miR167 regulatory pathway might be involved in male sterility as target genes of retrograde regulation to adapt to adversity in cytoplasmic-nuclear cross talk.

## Conclusions

Our study provides a comprehensive identification and comparative of the expression of miRNAs and their targets between male sterile line HBP and fertile line G1 + HBP in floral and reproductive developmental processes of *Citrus grandis*. The altered expression of miRNAs and the genes in miRNA regulatory pathway in a sterile line of HBP are demonstrated. Some miRNA expression showed significant difference, such as miR156, miR167, miR399 and miR827, which might be related to male sterility of G1 + HBP. It is proposed that miRNA regulatory networks may be involved in the citrus floral development and retrograde regulation in nuclear-cytoplamic interaction in citrus CMS.

## Methods

### Plant materials

Based on the results of paraffin section screening in a previous research [19], the floral buds of male sterile line G1 + HBP and its fertile line HBP were divided into three reproductive developmental stages (Fig. 1). HBP-1, HBP-2, HBP-3 referred to stage 1 (from floral bud differentiation to pollen mother cell formation), stage 2 (from the formation of tetrads to the release of microspores), stage 3 (from the development of young microspores to the release of mature pollens), respectively [20]. Floral buds of HBP and G1 + HBP were collected from at least 50 inflorescences which were harvested from two individual trees, after the phenotype of the floral buds in three stages was determined. The sampling was done by mixing the floral buds in three different stages according to the phenotypic difference (Fig. 1). Two biological replicates for the floral buds were performed, as HBP-1_1 and HBP-1_2 referred to the first and the second biological replicates respectively. Samples for each stage were immediately frozen in liquid nitrogen and stored at −80 °C until use. Total RNA was isolated using modified TRIzol method according to Liu et al. [62].

### Construction of small RNA and degradome libraries

Small RNA libraries for six samples were constructed. The small RNAs in size ranging from18 nt to 30 nt were purified from total RNA and ligated to 5’ and 3’ RNA adaptors. These RNAs were reverse-transcribed into cDNAs and then amplified by PCR to obtain sufficient sequencing products. Finally, the PCR products were subjected to Illumina sequencing on an Illumina Genome Analyzer at the Beijing Genomics Institute (BGI, http://www.genomics.cn/en/index).

One degradome library with mixed samples from six materials was constructed as previously described [37, 63]. PolyA RNA molecules were ligated to a 5’ RNA adaptor containing a *Mme* I recognition site. The ligation product was then reversed transcribed into double-strand cDNA and digested by *Mme* I and then ligated to a 3’ adaptor. The ligation products were amplified, followed by sequencing using Solexa technology at the Beijing Genomics Institute.

### Bioinformatic analysis

After the removal of low quality tags, 3’ adapter and adapter-adapter ligation products, clean tags ranging from 18 to 30 nt were obtained. These sRNAs were annotated into different classes such as rRNA, tRNA, small nuclear RNA (snRNA) and small nucleolar RNA (snoRNA) as mapped to Rfam (http://rfam.sanger.ac.uk/) and NCBI (http://www.ncbi.nlm.nih.gov/) database. Known miRNAs were searched by comparing sRNA tags with miRBase20 with a nearly perfect match (mismatch ≤ 2). The rest sequences were mapped to the genome of sweet orange (*Citrus sinensis*) (http://citrus.hzau.edu.cn/orange/) with perfect match to obtain their precursor sequences. To identify the putative precursors, ~450-nt fragments were extracted by extending 200 nt from the two ends of the small RNA mapping loci, respectively. The secondary structure was predicted by MIREAP (https://www.mireap.net/) program and the minimum free energy was set below −20 kcal mol − 1 was required. In addition, the miRNA* and miRNA pairs should satisfy the requirement of the 2 nt overhangs on the 3’ end as described previously [23].

### Stem-loop qPCR of miRNAs

The expression patterns of miRNAs in different samples were tested using stem-loop qPCR method [64]. cDNAs were reverse transcribed from total RNAs using SuperScript III Reverse Transcriptase (Invitrogen, USA). PCR was performed using 0.5 μl of cDNA products and 5 μl of SYBR GREEN PCR Master mix and 0.5 μM forward and reverse primers in a 10 μl total reaction system. The reaction program was performed on the ABI 7900 Real Time System (Applied Biosystems, USA) in a program at 50 for 2 min and 95 for 1 min, followed by 40 cycles at 95 for 15 s and 60 for 1 min. Two biological replicates in two years were conducted and U6 was used as the endogenous control [65]. The stem-loop primers were listed in Additional file 6.

### RLM-5’RACE

RNA ligase-mediated rapid amplification of 5’ cDNA ends was performed to validate the miRNA targets using the GeneRacer kit (Invitrogen, https://www.lifetechnologies.com). 5 μg total RNA was ligated to a RNA adapter by T4 RNA ligase. The ligation products were reverse transcribed, followed by amplification using a 5’ RNA adapter and a 3’ gene specific primer. A 3’ gene specific nested primer was used in a second PCR amplification if necessary. The final PCR products were gel purified and sequenced. The primers used in 5’ RACE were listed in Additional file 6.

### Northern blot of miRNAs

Northern blot was performed based on a method described by Liu et al. [30]. In brief, a hybond N+ nylon membrane (GE Amersham) with transferred total RNA was hybridized with Biotin 3’end labeled DNA oligo-nucleotide probe which was reverse complement of corresponding miRNA. The hybridization signal was detected using the Chemiluminescent Nucleic Acid Detection Module (Thermo Scientific). The primers of miRNA probes were listed in Additional file 6.

### Yeast one-hybrid assay

Yeast one-hybrid assay was performed using Matchmaker Gold Yeast One-Hybrid Library Screening System (Clontech, USA) according to the manufacturer’s instruction (http://www.clontech.com/). The promoter of miR167a was isolated from genomic DNA of HBP and cloned into the pAbAi vector. After linearization, the plasmid was transformed into the Y1H Gold yeast strain. *DREB* transcription factor gene was isolated and cloned into the pGADT7-AD vector, followed by transformation into yeast with promoter. The protein was considered to bind to the promoter sequence if the transformed yeast cells grew successfully on SD/-Leu/AbA medium plates.

### Dual-luciferase assay of transiently transformed *Nicotiana benthamiana* leaves

This experiment was performed according to the method described in Hellens et al. [66] with slight modification. The *DREB* transcription factor gene was isolated from cDNA of HBP and cloned into pH7GW2D binary vector. The promoter of miR167a was isolated from the genomic DNA of HBP and cloned into pGreenII 0800-LUC vector. They were transferred into *Agrobacterium tumefaciens* EHA105, followed by infiltration into young leaf of *N. benthamiana*. Three days after the inoculation, 3 mm leaf discs were cut and placed into a 384-well-plate. Firefly Luciferase and Renillia luciferase measurement were conducted using Dual -Luciferase System (Promega, USA) reagents according to the manufacturer’s instruction.

### Subcellular localization of DREB

The *DREB* transcription factor gene was isolated from cDNA of HBP and cloned into pM999-GFP vector (kindly provided by Oil Crops Research Institute of the Chinese Academy of Agricultural Sciences). Mesophyll protoplasts were isolated from 2-months-old HBP (*Citrus grandis*) and co-transformed with pM999-GFP-*DREB* and pM999-CFP-*GHD7* (kindly provided by Prof. Lizhong Xiong, Huazhong Agriculture University). Fluorescence in the transformed protoplasts was imaged using a confocal laser scanning microscope after incubation at 23 for 36 h.

## Additional files

Additional file 1: Information of identified known miRNAs in twelve libraries, including sequences, length, expression abundance of miRNAs and fold change and p value between HBP and G1 + HBP in three stages. (XLSX 105 kb) Additional file 2: Information of precursors of 184 known miRNAs and 22 novel miRNAs, including location, minimum free energy and sequences. (XLSX 29 kb) Additional file 3: Analysis of reproducibility of miRNAs expression data in two biological replicates for each sample and the expression profile of 14 selected miRNAs in sequencing data and qPCR in each biological replicates. (XLSX 241 kb) Additional file 4: Information of targets identified from degradome sequencing. (XLSX 16 kb) Additional file 5: Auxin level in HBP and G1 + HBP. The six columns on the X-axis refer to HB-1, G1 + HB-1, HB-2, G1 + HB-2, HB-3, G1 + HB-3, respectively. Y-axis indicates the relative level in HBP and G1 + HBP. Columns and bars represent the means and standard errors (*n* = 4). (TIF 505 kb) Additional file 6: All the primers used in this study including qRT-PCR, RLM-5’RACE and Northern blot. (XLSX 14 kb)
